# Detection of Power Line Insulators in Digital Images Based on the Transformed Colour Intensity Profiles

**DOI:** 10.3390/s23063343

**Published:** 2023-03-22

**Authors:** Michał Tomaszewski, Rafał Gasz, Jakub Osuchowski

**Affiliations:** Department of Computer Science, Faculty of Electrical Engineering, Automatic Control and Informatics, Opole University of Technology, Prószkowska 76 St., 45-758 Opole, Poland

**Keywords:** power insulator, object detection, signal classification, signal processing, image analysis, decision tree, random forest, XGBoost, Welch, Periodogram

## Abstract

Proper maintenance of the electricity infrastructure requires periodic condition inspections of power line insulators, which can be subjected to various damages such as burns or fractures. The article includes an introduction to the problem of insulator detection and a description of various currently used methods. Afterwards, the authors proposed a new method for the detection of the power line insulators in digital images by applying selected signal analysis and machine learning algorithms. The insulators detected in the images can be further assessed in depth. The data set used in the study consists of images acquired by an Unmanned Aerial Vehicle (UAV) during its overflight along a high-voltage line located on the outskirts of the city of Opole, Opolskie Voivodeship, Poland. In the digital images, the insulators were placed against different backgrounds, for example, sky, clouds, tree branches, elements of power infrastructure (wires, trusses), farmland, bushes, etc. The proposed method is based on colour intensity profile classification on digital images. Firstly, the set of points located on digital images of power line insulators is determined. Subsequently, those points are connected using lines that depict colour intensity profiles. These profiles were transformed using the Periodogram method or Welch method and then classified with Decision Tree, Random Forest or XGBoost algorithms. In the article, the authors described the computational experiments, the obtained results and possible directions for further research. In the best case, the proposed solution achieved satisfactory efficiency (*F1 score* = 0.99). Promising classification results indicate the possibility of the practical application of the presented method.

## 1. Introduction

In many areas, overhead power lines are mainly used to transmit electricity, which means that they are also one of the key elements of each country’s security. The overhead line comprises three essential components: conductive wires, transmission towers, and power insulators. Overhead line insulators have two main functions: they isolate the conductor from the ground and tower structure and provide mechanical support for the conductive wires. Insulators used in overhead lines, particularly high-voltage lines, require regular inspection. It is necessary due to their degradation through the influence of weather conditions, the impact of temperature, as well as high voltage and mechanical stress. Because electricity transmission is crucial for the industry, economy and defence of the state, power outages caused by overhead line failures can have catastrophic consequences for the entire country. The safety and stability of high-voltage lines may also significantly impair the conditions and comfort of the life of citizens. To prevent electricity supply interruptions and reduce their duration to an absolute minimum, electricity distribution companies should conduct regular detailed and routine inspections of overhead lines [1]. Although in the field of automatic inspection of high-voltage lines, numerous research works are still being carried out [2,3,4,5,6,7,8,9], there are still some shortcomings and challenges that need to be solved. To create a smart power grid, it is necessary to propose methods for automatic detection of internal thermal defects, external problems such as foreign objects and damage to the power transmission equipment in time [2].

The power infrastructure is of key importance for the proper functioning of the economy, both industry and services, and for the stable functioning of households. Unfortunately, like any system, the power grid is subject to the ageing process, which may result in unforeseen interruptions in its functioning. The first step in the process of visual diagnostics of high-voltage lines is always the detection of its key elements [6,7,8,10,11]. The traditional manual inspection methods are usually not fast enough and often do not provide sufficiently detailed detection data [2,9]. If a failure or damage to key line components is not detected in time, it can lead to a local, or even global blackout [9,12]. The occurrence of a blackout can cause significant financial losses and, in extreme cases, a humanitarian disaster. Many authors [2,10,13,14,15,16,17] see the solution to this problem in the use of UAVs and broadly understood methods of visual inspection.

It is necessary to develop new, fast methods of detecting insulators in digital images [18], which will be part of a more extensive diagnostic process to assess their condition to avoid severe power grid failures. The methods should be able to be implemented on UAVs, which necessitates the development of fast and computationally simple algorithms. However, in [19], authors state that the deep learning-based object detection technology not every case can be used in UAV transmission line inspection to achieve efficient and accurate detection because of its complex structure and the demand for a large amount of computing performance.

This paper aims to present the developed method of detecting power line insulators on digital images using selected signal analysis methods. The insulators detected in the images can then be subjected to further in-depth evaluation regarding their proper operation. In the study, the authors used the data set they collected (for the data set see the attached Supplementary Materials), consisting of images obtained during an Unmanned Aerial Vehicle (UAV) flight along a high-voltage line. The starting point for the conducted analyses was the results of the research described in the publication [20], presenting the method of describing objects on digital images based on analysing the frequency of specific points. As a result of its application for the constructed data set, a set of points characteristic for insulators of transmission lines for each digital image was obtained. Then, it is verified whether a specific point is located on the insulator or outside it. The lines were generated between individual characteristic points along which colour profiles were determined. These profiles were transformed using selected digital signal processing methods, and then the classification of these signals was classified using the following machine learning algorithms: Decision Tree, Random Forest, and eXtreme Gradient Boosting (XGBoost) classifier.

## 2. Background

Various types of insulators are subject to different types of failures. The faults of polymer insulators differ significantly from those of porcelain and glass insulators. Many factors can lead to the failure of insulators, for example, manufacturing defects, incorrectly selected insulators, vandalism, harsh working conditions, pollution or the influence of weather conditions. The most common failures of overhead line insulators include, among others, cracking of insulator discs due to expansion of the cement used to connect the plate to the cap, hook or shaft [21,22,23].

### 2.1. Visual Recognition and Fault Detection of Power Line Insulators

It is estimated that 50% of the overhead line maintenance costs are related to insulators diagnostics, replacement and repair. Insulator failures cause about 70% of downtime in line operation [1]. This picture shows the importance of diagnostics and early detection of insulator failures. Diagnostic tests of insulators serve three main tasks: identification of damaged insulators and those posing a high risk of failure, assessment of the degree of ageing degradation of the properties of insulators, and detection of poorly made or incorrectly selected insulators. Insulator diagnostics generally can be divided into two different types: tests in laboratory conditions and inspections of overhead lines [24]. Condition inspections of insulators during operation are essential because today’s electricity demand puts enormous pressure on electricity distributors to minimise the downtime of the overhead lines. Assessing the condition of power lines is the basic activity that directly impacts reducing the number of failures in distribution networks. From the power system security viewpoint, high and extra-high voltage lines have strategic importance.

Collecting diagnostic information about power lines can be divided into:

- actions performed from the ground by human walking teams and off-road vehicles;
- aerial operations with the use of helicopters and UAVs [15,25,26];
- activities performed by devices mounted directly or close to power line elements, for example, various types of monitoring systems and diagnostic robots [27,28,29,30].

The dynamic development of various types of vision systems used for the observation of public spaces and the protection of various types of industrial facilities allows for their simple application in the electrical power industry in combination with computer image analysis techniques. Recognising the condition of electricity infrastructure objects in digital images creates a wide range of potential applications. These may include [29,31,32,33]: precise measurements of the position of line structural elements, monitoring to support early warning systems, condition monitoring, and fault localisation in high-voltage networks, identification of line hazards arising from the immediate environment. The data collected during a ground inspection are usually images obtained using various cameras. Their interpretation is complicated, as they are usually taken from large angles and considerable distances. This is due to the specificity of overhead line construction—the most critical components, for example, wires, insulators, and connectors, are suspended over a considerable height, as it is crucial to isolate the conducting wires from the ground. The data obtained during helicopter or aeroplane overflights allows a more accurate analysis of the essential elements of the line, as such images are usually taken from observation points suspended directly above the line. On the other hand, using UAVs allows line elements to be analysed from virtually any angle and relatively short distances. The use of UAVs means that the inspection of an overhead line can be more efficient and accurate, as the drone can fly closer to the line than a helicopter or foot patrol [34].

In addition, inspections carried out by unmanned aerial vehicles allow for a significant reduction in the cost of the entire process and shorten the time needed to perform the diagnostics of the overhead power line. Currently, there are more and more different flying platforms offering different possibilities of imaging objects (i.e., resolutions and types of imaging). An interesting option is also the possibility of using a swarm of drones, which allows for the simultaneous imaging of overhead lines by several vehicles from different perspectives. The problem is the massive amount of data received, which must be analysed manually afterwards [31]; therefore, it is necessary to create new efficient methods of their processing.

### 2.2. Detection of Power Insulators—A Bibliography Review

The paper [35] presents a simple method for detecting insulators in aerial photographs by binarising the image and applying morphological operations. However, the detection is limited to tempered glass insulators only, and the criteria for selecting the adaptive threshold for different lighting conditions are not included in this paper. Other work [36,37] has suggested using colour features extraction to detect insulators. In work [38], colour-based segmentation was used to separate the insulator from the background.

In publications [39,40], edge-based feature extractors have been used to detect porcelain insulators from images taken with drones and cleaning robots. The methods presented performed poorly on images where the background was not uniform. A publication by Zhao et al. [41] proposed an insulator lattice model by grouping similar appearance glass and porcelain insulator components together and then performing a network search using the Markov Random Field (MRF) algorithm. The extracted data are then combined with spatial contextual information to localise multiple insulators quickly. The proposed method works stably on a complex background, but its performance is only guaranteed when a group of insulators appear together in an image, which significantly limits its application.

Liao and An in [42] proposed a robust Multiscale and Multi-Feature (MSMF) descriptor based on local features. On the other hand, in [43], Haar features and the AdaBoost classifier were used to detect the insulator. In this paper, a synthesised 3D model of the insulator was used to train the classifier. It showed a significant improvement in detection accuracy. However, both methods [42,43] only detect insulators in images taken from a long distance with low resolution, which is unsuitable for further defect analysis.

Li et al. in [44] used Vertical Profile Projection curves (VPP) as features to determine the shape of insulators and a Support Vector Machines (SVM) classifier to detect them. Wang et al. [34] proposed a Gabor feature detector and SVM classifier for insulator detection. The methods proposed in [34,39] are based on a repeating pattern on the insulator. However, insulators are only well observed when a picture is taken perpendicularly to a power line. As a result, this method’s photo of the insulator taken at any angle is not usable. Li et al. [45] proposed using a local and global relevance map to segment insulators. However, their method only works when the texture and intensity of the background and foreground areas are clear. Such a condition usually occurs only when aerial photographs of insulators are taken from closer distances and using appropriate camera optics.

The method developed in [46] based on Speeded Up Robust Features (SURF) and Intuitionistic Fuzzy Set (IFS) algorithms allows localising in aerial photographs without using pattern and segmentation. The first step searches for crucial features using the SURF algorithm. Then the obtained points are divided into a certain number of classes using the IFS algorithm based on the correlation coefficient. If the correlation between the obtained sets is more significant than the set value, then both classes can be treated as sets of the same class. The insulator is identified based on characteristic shape factors values such as slenderness or duty ratios. Another approach is to locate an insulator based on colour, an example being the research published in [47]. Methods based on the SIFT and SURF algorithms can locate an object accurately; however, their application has some limitations. Depending on the complexity of the background, they generate large numbers of significant (local features), which translates into increased computational costs. In the case of aerial photographs, insulators are most often located against very different backgrounds. A previously created pattern is also most often required to locate the feature.

Oberweger et al. [48] presented a novel approach for detecting insulators in aerial photographs. They based their algorithm on discriminative training of local gradient-based feature descriptors and a voting scheme based on the Random Sample Consensus (RANSAC) algorithm. However, their algorithm does not allow the detection of multiple insulators in a single image.

Another different approach to trying to locate an insulator but using pattern matching is the approach presented in [49]. In this paper, the author segments the image into specific classes using Statistical Region Merging (SRM) and then converts the image to greyscale for histogram analysis. The histogram at this stage represents the individual objects in the image. Insulator identification is based on pattern matching using the correlation method. However, this method cannot cope with irregularities in the insulator structure and is sensitive to noise in the image.

Jabid and Uddin [50] used a classical detection method based on a sliding window allowing the detection of Local Directional Pattern (LDP) features and an SVM classifier. Their method not only needs to scale the input image to multiple sizes but also rotates the input image in multiple orientations to account for changes in size and rotation, which significantly slows down the detection process.

In [51], the authors used a cascaded CNN architecture based on Reverse Polish Notation (RPNs). This work combined VGG Neural Networks and ResNet networks, but the limitation is the insufficient speed for real-time operation. In the paper [35], a convolutional neural network is used for insulator feature extraction and classification, and OTSU-based segmentation is applied in the next step. In [52], a deep learning algorithm based on feature detection is proposed. Region Proposal Network (RPN) is used to generate region proposals, and a Fully Convolutional Network (FCN) is used to obtain object maps.

The above methods were tested on images taken with aircraft and helicopters. As these vehicles have several disadvantages, for example, high operating price, complicated operation, and susceptibility to bad weather conditions (e.g., strong wind), insulator detection systems based on ground vehicles have also been proposed [40,50,53,54] Li et al., in their paper [40], used an improved MPEG-7 edge histogram descriptor to detect insulators on frames from videos taken from the ground. The publication [54] used a detector based on a wavelet transform and an SVM classifier to detect insulators. Another publication by the same authors [53] extracted features using a wavelet transform and then used a hidden Markov model to classify damaged insulators.

## 3. Materials and Methods

### 3.1. Description of Proposed Method

**Figure 1:**
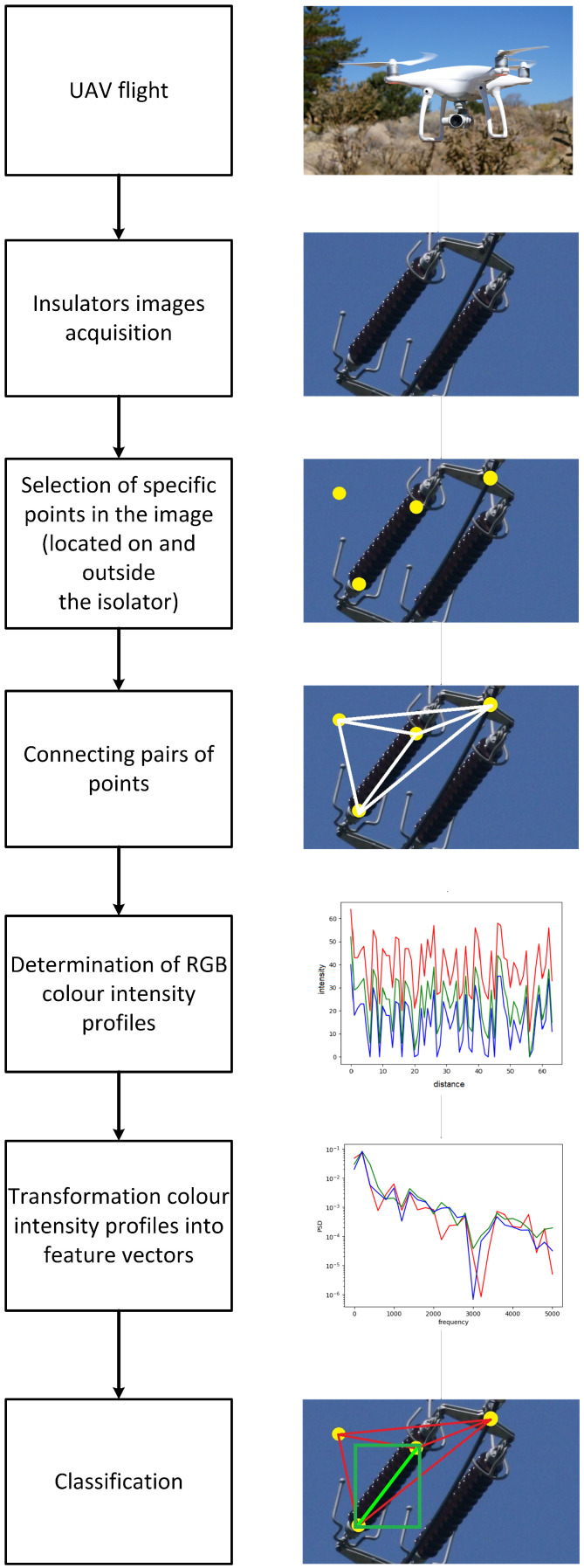
Consecutive steps of the proposed method.

The following stages of the conducted research experiment are presented in Figure 1. The starting point was to carry out several drones and power line flights and registration of video material in the form of digital images and films.

Then, a selection and preprocessing of images showing electrical insulators were performed. The next part of the paper describes the data set built for this research in detail. When analysing the appearance of insulators, regardless of their type, each has a characteristic structure and colour. The colour and structure affect the colour characteristics of the insulator in the painting, especially against the background of the sky, greenery or other infrastructure. The next step of the described method is determining specific points on the analysed digital image, which may be located both on the insulator and outside it. For this purpose, the method presented in the paper [20] was used, which consists of selecting characteristic local features in the images to detect specific objects of the power line in various scenes. The research described in the paper aimed to automate searching for characteristic objects in digital images with a limited training set. In the conducted research, the description of the insulator was made based on a set of distinctive local features, which were characterised by the highest repeatability in individual images of the test set. The SURF algorithm was used to search for and describe local features.

However, the applied method has limited efficiency, and not all the points found are located on the insulator. The method described in this paper consists in properly checking whether a given pair of points is on the insulator or outside it. The proposed method determines the characteristics of the colour change analysis of a specific line between two points (the colour profile). The colour characteristic of the line within the insulator has some peculiarities that distinguish it from the characteristics of the colour profile generated along with other elements of the environment or surroundings and only part of the insulator.

Therefore, in the proposed method, in the next stage, the process of connecting the previously determined points with lines for which the colour profile (RGB components) was calculated along the designated line was performed. This way, feature vectors of different lengths were obtained for each pair of points. Then, each determined vector was transformed according to the selected method into a feature vector of the same length resulting from the used parameters.

Two transformation methods were used to obtain the characteristics. They relied on frequency changes into a set of features, namely the transformation by the Welch method and the Periodogram. The Cross Spectral Density (CSD) method was also checked, obtaining similar results in the described case. Therefore, it was omitted in this paper. Welch’s method provides a consistent estimate of the power spectral density [55]. Thus, the data segments can be represented as

(1) $$\begin{array}{c} x_{i}\left(n\right)=x\left(n+iD\right)\\ n=0.1,\ldots ,M-1\\ i=0.1,\ldots ,L-1 \end{array}$$

where iD is the starting point for the *i*th sequence. Observe that if D=M, the segments do not overlap, and the number *L* of data segments is identical to the number *K* in the Barlett method. However, if D=M/2, there is a 50% overlap between successive data segments, and L=2K segments are obtained. Alternatively, *K* data segments each of length 2M can be formed. The second modification made by Welch to the Bartlett method is to window the data segments prior to computing the Periodogram. The result is

(2) $$\begin{array}{c} P{xx}^{\left(i\right)}\left(f\right)=\frac{1}{MU}{\left|\sum_{n=0}^{M-1}xi\left(n\right)w\left(n\right)e^{-j2\pi fn}\right|}^{2}\\ i=0,1,\ldots ,L-1 \end{array}$$

where *U* is a normalization factor for the power in the window function and is selected as

(3) $$U=\frac{1}{M}\sum_{n=0}^{M-1}w^{2}\left(n\right)$$

A Periodogram is a kind of discrete Fourier transform. According to its definition, the Periodogram can be expressed as follows: let the function f(t) be

(4) $$\frac{T}{2}a=\int_{t_{1}}^{t_{1}+T}f\left(t\right)cos\left(kt\right)dt$$

(5) $$\frac{T}{2}b=\int_{t_{1}}^{t_{1}+T}f\left(t\right)sin\left(kt\right)dt$$

where *T* can be chosen equal to an integer multiple $\frac{2\Pi }{k}$. A Periodogram is used as an estimator in spectral analysis (e.g., statistical analysis of data, description of signal strength, and others). The results are often burdened with a significant error, but it is used quite often. Usually, practice shows that it is typically adequate for clearly periodic functions. However, as presented later in the paper, it can also be used effectively in other cases. In the Periodogram, the waveform is approximated as the sum of the sine waves. The frequencies of these waves are multiples of the reciprocal of the analysed sample duration.

**Figure 2:**
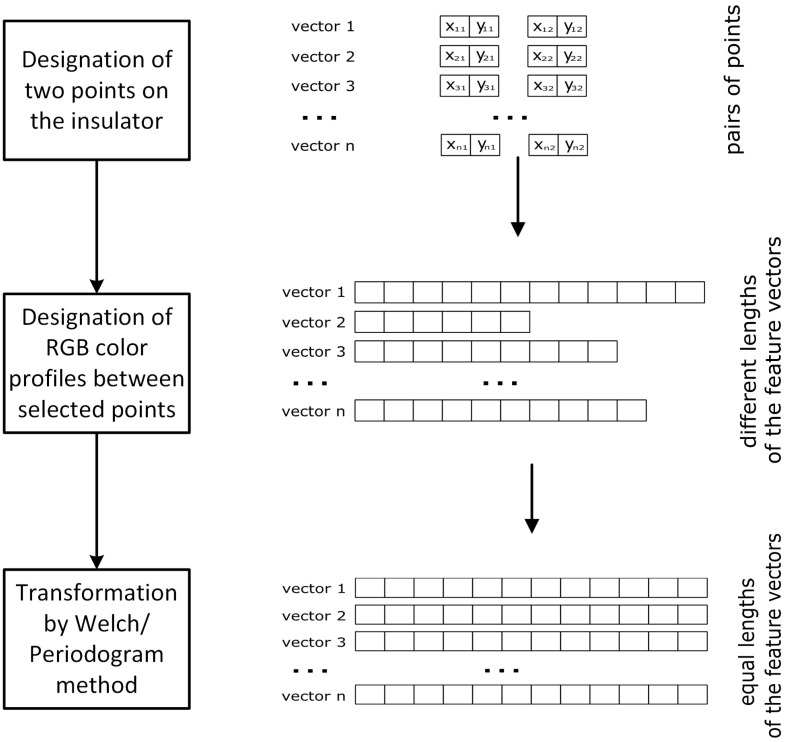
Transformation of the colour intensity profiles determined between individual points (vectors with different lengths depending on the distance between pairs of points) into feature vectors of equal length.

The described transformation procedure is shown in Figure 2. It allowed for the transformation of a set of intensity colour profiles into feature vectors of the same length, which contain a spectral density estimation (characteristic frequencies occurring in the analysed signal). In the next step of the described method, selected machine learning algorithms were used to classify all given feature vectors. In the classification process, it was determined whether the vector (originally, colour intensity profile) is located on the insulator or outside of it.

Additionally, the duration time of each part of a method for various configurations of input parameters was analysed.

### 3.2. The Data Set Used in the Research Process

**Figure 3:**
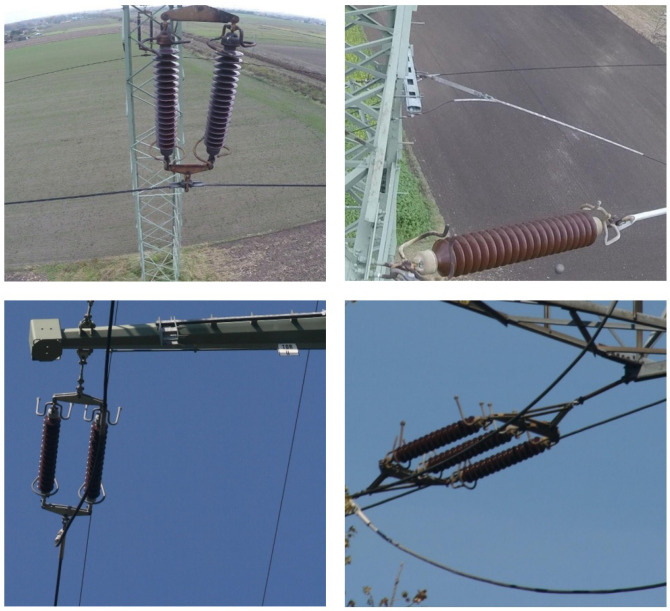
Example images of power line insulators from the prepared data set.

The research was carried out with a self-created data set, which consisted of 116 images of insulators of various types taken in multiple kinds of surroundings (examples is shown in Figure 3). The images of the analysed insulators were taken on real objects in the southern part of Poland. In the images, apart from isolators, there are other background elements such as sky, clouds, elements of equipment, trusses, farmlands (in various stages of vegetation growth), grass, tree branches, and leaves.

The images show the points between which the colour profiles were drawn, following the method described earlier in the paper. As a result of determining profiles between all designated points, files were obtained containing data with colour profiles for points located on the insulator and data for colour profiles between points located outside the insulator. Finally, after transforming the signals, the balanced data set contains 3219 files: 1473 feature vectors with the colour profiles located on the insulator (46%) and 1746 feature vectors with the colour profiles located outside the insulator (54%).

### 3.3. Classification Methods Used in the Research Process

Various classification methods were analysed during the study, but finally, it was decided to present the analysis for classifiers that are an extension of the idea of decision trees, for which the most promising results were obtained. The first was the Decision Tree classifier, a supervised machine learning algorithm that uses a set of rules to make decisions, similar to how humans make decisions. Another algorithm was the Random Forest algorithm, a meta estimator that fits several decision tree classifiers on various sub-samples of the data set and uses averaging to improve the predictive accuracy and control over-fitting. The last of the algorithms used to classify the signals obtained at the previous research stage was the eXtreme Gradient Boosting algorithm-an advanced ensemble learning method.

The Decision Tree classifier was only a reference point, and no selection of hyperparameters was made for it, in contrast to the two subsequent algorithms. After performing the hyperparameters tuning process, Random Forest classifier parameters have been set to the following values:

- the maximum depth of the tree (max_depth) was set to 5,
- the minimum number of samples required to be at a leaf node (min_samples_leaf) was set to 2,
- the minimum number of samples required to split an internal node (min_samples_split) was set to 3, and all other parameters were left in the default position.

The XGBoost classifier parameters have been set to the following values:

- the maximum depth of a tree (max_depth) was set to 5,
- the minimum loss reduction required to make a further partition on a leaf node of the tree (gamma) was set to 5, the step size shrinkage used in the update to prevent overfitting (eta) was set to 0.075,
- the number of gradients boosted trees (n_estimators) was set to 1000, the L1 regularization term on weights (alfa) was set to 0.001, the L2 regularization term on weights (lambda) was set to 0.01, and all other parameters were left in the default values.

The parameter analysed in each case of the feature vectors transformation from colour profiles of variable length into feature vectors of equal, fixed length was the nfft parameter (Nonequispaced Fast Fourier Transform) which is the length of the FFT used. In the case of the described studies, the value of the nfft parameter was set from 64 to 1280 in increments of 64. Increasing the size of the nfft provides higher frequency domain resolution at the expense of lower time-domain resolution. Despite the many advantages of the methods used in the research, they also have some disadvantages, such as the speed of processing, the need to train the network, and the size of the data set necessary to obtain good results.

The tests were carried out on a computer equipped with an Intel i7-9700k processor, 16 GB of RAM memory, a Geforce RTX 2700 graphics card, and a Microsoft Windows 10 Pro operating system. The computing environment chosen for the research was Python 3.10, with the necessary libraries.

During the study, the primary indicator adopted as a measure of the algorithm’s effectiveness was the *F1 score* indicator, which is the harmonic mean of precision and recall. The *F1 score* parameter can be calculated using the formula:

(6) $$\;F1\;score=2*\frac{Precision*Recall}{Precision+Recall}$$

where:

- Precision is a parameter that says how many of all predictions made by the classifier are correct,
- Recall is a parameter that informs how many of all marked objects have been correctly classified.

During the analyses, the parameter weighted-averaged *F1 score*, calculated by taking the mean of all per-class *F1 scores* while considering each class’s support, was examined. Support refers to the number of actual class occurrences in the data set.

## 4. Results

**Figure 4:**
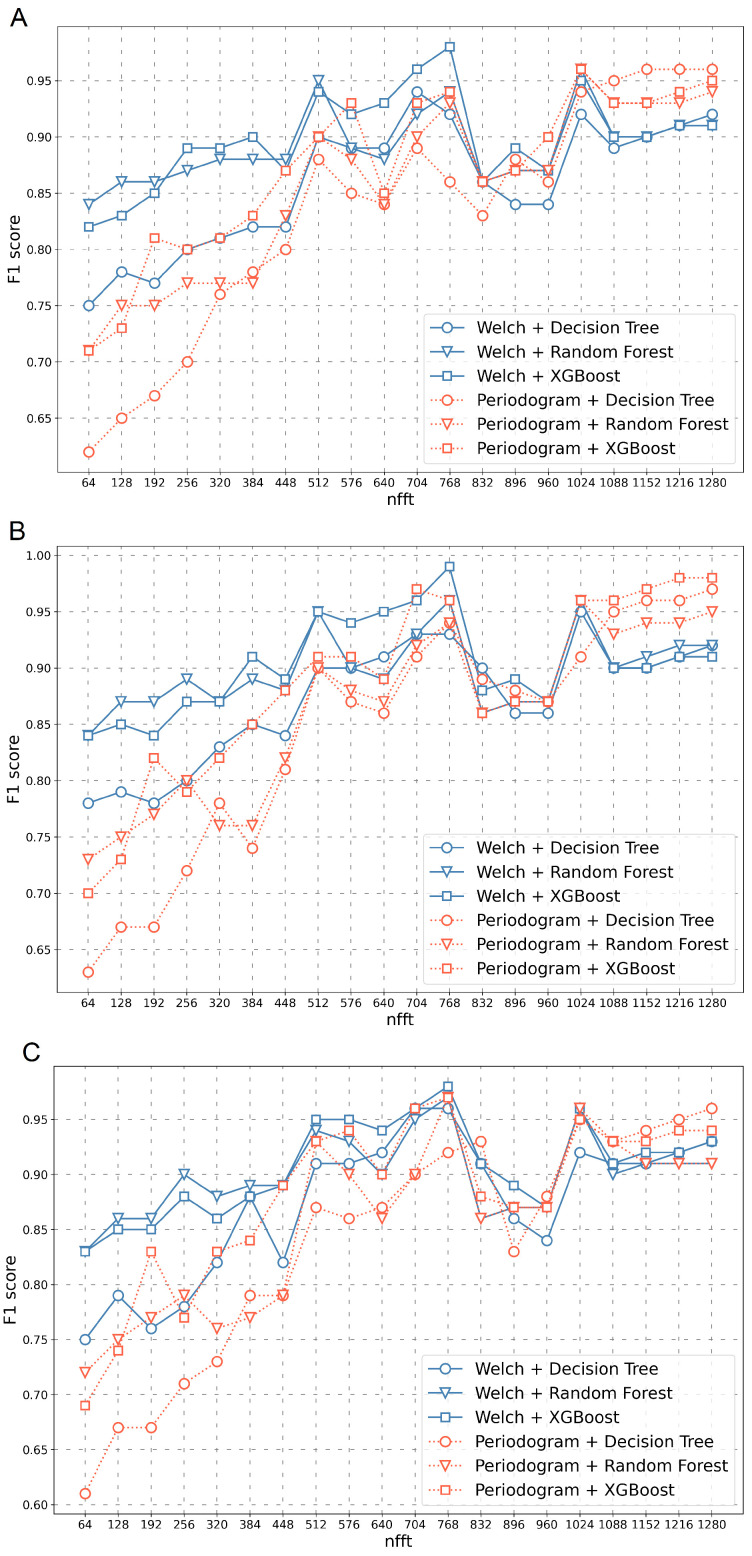
The value of the *F1 score* parameter depending on the nfft value for various transformation methods and classifiers as well as three channels analysed separately (Red—(**A**), Green—(**B**), Blue—(**C**)).

Initially, the value of the *F1 score* measure was compared separately for each RGB channel (Figure 4).

**Figure 5:**
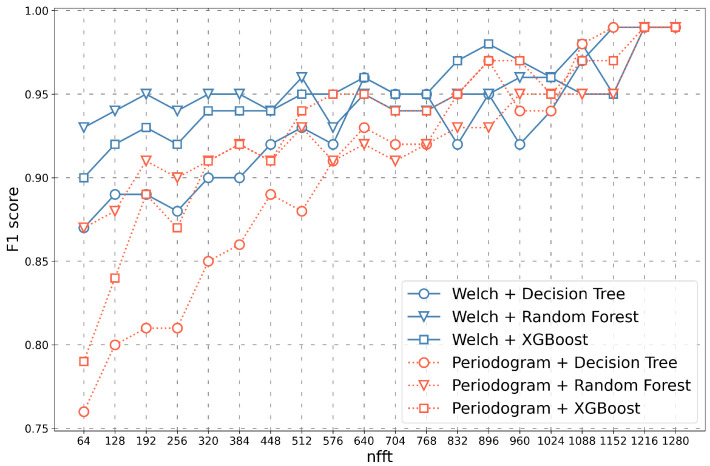
The value of the *F1 score* parameter depending on the nfft value for various transformation methods and classifiers-results obtained for three connected channels (R + G + B).

The value of the *F1 score* depending on the length of the feature vectors obtained for different transformation methods (Periodogram, Welch) as well as different classifiers (Decision Tree, Random Forest, XGBoost) are presented in Figure 4. The figure shows three channels separately (Red—A, Green—B, Blue—C). Classification results for three connected channels (R+G+B) are shown in Figure 5. Studies have been conducted for the nfft parameter, starting from the length of 64 and ending with the length of 1280.

As demonstrated in Figure 4, for each channel separately, the value of the *F1 score* increases, provided that the nfft parameter increases. The highest *F1 score* value was obtained for the Green channel (Figure 4B). In each of the presented cases, a decrease in the *F1 score* in the range of the nfft parameter from 832 to 960 can be observed.

As shown in Figure 5, the results vary significantly depending on the type of transformation method and the selected classifier. For small nfft parameters, the Welch method transformation in combination with the Random Forest classifier performed best, and the Periodogram method transformation in combination with the Decision Tree classifier performed worst. For larger nfft values, all results remained at *F1 score* = 0.99. Moreover, it should be noted that in the case of this graph, the dependencies presented on it were more similar to linear than in the graphs shown in Figure 4, for example, for three RGB channels separately. The method worked more stably in this case and achieved better results even for smaller values of the nfft parameter.

During the research, the method’s execution time was also analysed: time of the feature vectors transformation (Welch and Periodogram), time of learning classifiers (Decision Tree, Random Forest, XGBoost), and time of the signal classification.

The transformation time of the feature vectors changes linearly in proportion to the size of the nfft parameter–the larger the parameter, the longer the transformation time. For the Periodogram algorithm, the transformation time was within the range of 10.89 s to 14.64 s, and for the Welch algorithm in the range from 11.96 s to 18.89 s.

**Figure 6:**
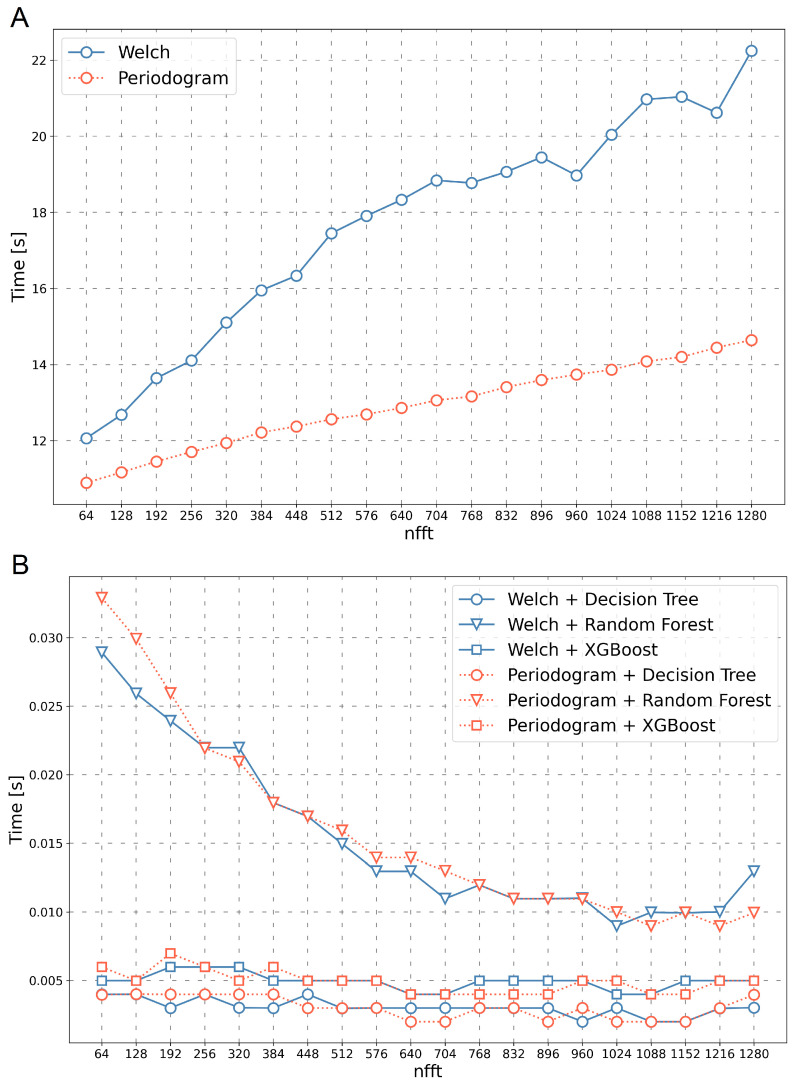
Transformation time (**A**) and classification time (**B**) depending on nfft values for various transformation methods and classifiers.

The classification time ranges from 0.0019 s to 0.032 s, as shown in Figure 6B. The choice of the transformation method has only a tiny impact on the classification time. It can also be observed that the Random Forest algorithm has the longest classification time, which gradually decreases as the length of the feature vector increases. For other algorithms (Decision Tree and Random Forest), no significant effect of the length of the feature vector on the classification time was observed.

**Figure 7:**
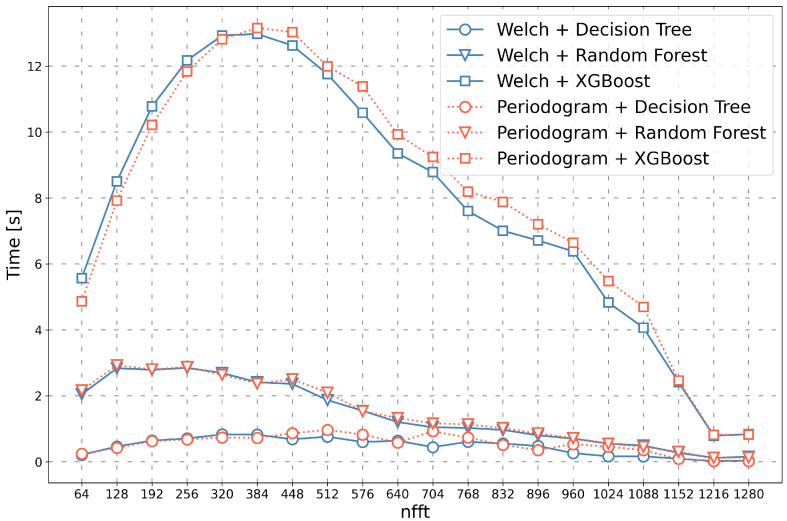
The learning time of classifiers depends on the nfft values for various transformation methods and classifiers.

The learning time of selected classifiers is shown in Figure 7. It can be noticed here that the choice of the transformation method has only a slight impact on the learning time of classifiers. The learning time of the XGBoost algorithm is initially much higher than the other algorithms, with a maximum of 13.15 s. For all algorithms, the learning time initially increases and eventually decreases to less than 1 s. In the authors’ opinion, increasing the nfft parameter value allows the classifiers to more easily distinguish observations from one another and assign them to individual classes. This is also confirmed by the increase in the classification efficiency along with the increase in the nfft parameter Figure 6.

The analyses have shown that the most significant impact on the execution time of the entire algorithm has the time of the feature vectors transformation, which increases provided that the size of the nfft parameter rises as well. Nevertheless, it seems intentional to set the parameter to a higher value (in the range from 1152 to 1280) because it significantly reduces the time of learning classifiers and time of classification and increases the effectiveness of the presented method (value of the *F1 score* = 0.99).

## 5. Discussion

The main objective of the study was to develop a novel method for the automatic detection of power line insulators in digital images. This method should be possible to implement on UAVs and, as a result, should allow for improving the insulator’s diagnostics process. According to [56], in recent years, with the wide application of UAVs in electric power line inspection, inspection efficiency has been promoted rapidly. Likewise in [57,58,59,60,61] were presented arguments for the various possibilities of using UAVs in the power lines diagnostics. However, some publications [62,63,64,65,66,67] indicate problems connected with this technology. The most important ones were described in [68], there are

- The degree of autonomy of the inspection flight;
- Flight control stability issues—an inspection flight robot in response to the complex inspection environment has difficulty in achieving high precision and stable hovering;
- UAV battery replacement issues;
- Inspection data fault detection (low accuracy).

Known in the literature, methods of detecting insulators mentioned in the theory section (e.g., methods based on the SIFT and SURF algorithms, methods based on convolutional neural networks are vulnerable to the changing of lightning condition, background, camera angle, perspective, and foremost they are difficult to implement on UAVs board due to the high demand in terms of the required computing performance.

Previously mentioned methods of detecting insulators in digital images achieve the results presented in Table 1 (omitted publications with a qualitative description of the detection efficiency).

For the presented methods, different ways of evaluating the effectiveness (metrics) and different data sets (test images of power line insulators) were used. This makes a direct comparison of the presented solutions difficult. In most cases, precision and recall metrics were used. Because the *F1 score* is the harmonic mean of these two indicators, it can be concluded that the method described in the paper achieves as high results (*F1 score* = 0.99) as several of the most effective methods listed in the quoted list. Therefore, it can be assumed that the method will be equally effective in practical applications. However, it is necessary to conduct research on an extended data set, taking into account a larger number of analysed cases.

The research was carried out using images taken during several drone flights using the same device (Matrice 210 rtk v2 with the Zenmuse x5s camera). It is necessary to verify the presented method under various conditions, for example, illumination, weather conditions, and the appearance of additional objects in the background. It is necessary to collect the extended vision data using various cameras to do this. The authors also have a thermal and hyperspectral camera, and the acquisition of this type of digital image is planned.

The method described in the paper was developed for a specific purpose: detecting insulators of power lines. However, the proposed method can also be used to detect, classify or segment other objects in digital images, which requires the construction of appropriate training data sets. Indeed, some objects with characteristic features (colour, shape, share of individual colour components) will be recognised more effectively than others, but this requires in-depth research.

Classifications of digital signals were made using a few selected machine learning algorithms: Decision Tree, Random Forest, and XGBoost. This solution was chosen to minimise the individual models’ training time and shorten the insulators’ classification time. This approach allows using the presented method when detecting insulators during a flight along a power line. Using more sophisticated algorithms based on artificial neural networks in the described process is possible, but it will probably significantly extend both the model learning and classification process. This approach also requires building a sufficiently large set of training data.

## 6. Conclusions

Satisfactory results of the developed method were obtained. In the best case, the proposed method achieved satisfactory efficiency with *F1 score* = 0.99. The value of the *F1 score* parameter increased along with the nfft parameter, for example, the length of the transformed intensity colour profile vector. The highest efficiency was obtained for the feature vectors created on joint all colour channels (R+G+B). However, after analysing each channel separately, it can be concluded that the *F1 score* achieved the highest classification result for the Green channel.

For short lengths of the feature vectors, the best results were obtained for the Welch signal transformation method in combination with the Random Forest classifier and the worst for the Periodogram transformation in variety with the Decision Tree classifier. It can also be noticed that the Random Forest algorithm has the longest classification time, which gradually decreases as the length of the features vector increases. In the case of the remaining algorithms (Decision Tree and Random Forest), no significant effect of the feature vector length on the classification time was found. The qualification times ranged from 0.0019 s to 0.032 s.

It can be noticed here that the choice of the feature transformation algorithm has little influence on the learning time of the classifiers. For the Periodogram method, the transformation duration ranged from 10.89 s to 14.64 s, and for the Welch method from 11.96 s to 18.89 s. The analysis shows that the transformation time of the feature vectors significantly affects the execution time of the whole algorithm.

The presented method has some limitations. The described experiment was carried out only for a set of insulators with similar construction; therefore, more tests should be performed for different types of insulators; however, it requires building an appropriate data set, which is time-consuming and expensive. Digital images of insulators were recorded under certain specific conditions; hence it is necessary to check the efficiency of the described method in many other conditions (types of lighting, backgrounds, seasons and geographic locations, changing weather conditions, etc.). A separate challenge is the detection of insulators in real time. It is worth mentioning that the operation time of the proposed method was one of the main assumptions of its development. However, the ultimate achievement of this assumption requires further tests on various computing platforms.

The method can be applied to other types of facilities, particularly other power system facilities, supporting various techniques for optimizing their operation (e.g., [69]). Nevertheless, it probably won’t be as effective as in the analysed case for objects with less regular shapes.

Only non-occluded insulators were analysed in the tests. Regarding the detection of occluded objects is a known problem of advanced digital image processing, it is also advisable to take this issue into account during the practical implementation of the proposed method.

Possible directions of further research include an analysis of the proposed method’s effectiveness on a more extensive set of data or an attempt to generate an artificial grid of points located on the insulator and beyond it, as well as an analysis of the effectiveness of such an approach depending on diverse generation variants. The authors also consider the concept of generating a synthetic mesh of points using an appropriate optical system. The method presented in the publication [70] uses a high-power laser to create an artificial grid of points on the observed object with a specific electromagnetic wavelength. After the correct detection of the laser points in the analysed image, all possible colour profiles are determined between them, which can then be classified using the method described in this paper.

During the research, an analysis of the method’s effectiveness was performed for the RGB colour space-it seems advisable to perform the analysis for other colour spaces, for example, HSL, HSB, CIELAB, or CIELUV. One of the relevant directions for further research is to conduct an attempt to detect and classify faults in the insulators. Building an appropriate, structured data set is necessary for such an attempt to be possible. The authors already have the proper material at their disposal, and work is currently underway to create a relevant catalogue of typical failures of power line insulators.

## Figures and Tables

**Table 1:** Effectiveness of methods described in the section “Detection of power insulators—a bibliography review”.

Method	Metrics	Source
Defects detection of glass insulator based on color image	Accuracy = 0.98	[37]
Self-shattering defect detection of glass insulators based on spatial feature	Precision = 0.92	[38]
A robust insulator detection algorithm based on local features and spatial orders for aerial images	Precision= 0.87	[42]
A method of insulator detection from aerial images	Precision = 0.96	[43]
A method of insulator detection from video sequence	Accuracy = 0.95	[44]
A method of insulator detection from aerial images	Accuracy = 0.92	[43]
Visual recognition and fault detection for power line insulators	Precision = 0.33	[48]
Vision diagnostics of power transmission lines: an approach to recognition of insulators	AUROC = 0.65	[49]
Rotation invariant power line insulator detection using local directional pattern and support vector machine	Precision = 0.89	[50]
Detection of power line insulator defects using aerial images analysed with convolutional neural networks	Precision = 0.79	[51]
Presented method	*F1 score* = 0.99	-

## Data Availability

A data set containing the transformed colour intensity profiles has been attached to the article. The data set has been divided into two classes: insulator (i), not insulator (ni).

## Supplementary Materials

The following supporting information can be downloaded at: https://www.mdpi.com/article/10.3390/s23063343/s1.

## Abbreviations

The following abbreviations are used in this manuscript:

AUROC	Area Under the Receiver Operating Characteristics
CIELAB	CIE 1976 L*, a*, b* colour space
CIELUV	CIE 1976 L*, u*, v* colour space
CNN	Convolutional Neural Network
CSD	Cross Spectral Density
FCN	Fully Convolutional Network
FFT	Fast Fourier Transform
HSB	Hue, Saturation, Brightness
HSL	Hue, Saturation, Lightness
IFS	Intuitionistic Fuzzy Set
LDP	Local Directional Pattern
MRF	Markov Random Field
MSMF	Multiscale and Multi-Feature
nfft	Nonequispaced Fast Fourier Transform
RANSAC	Random Sample Consensus Algorithm
RPN	Region Proposal Network
RPNs	Reverse Polish Notation
SIFT	Scale-Invariant Feature Transform
SURF	Speeded Up Robust Features
SRM	Statistical Region Merging
SVM	Support Vector Machines
UAV	Unmanned Aerial Vehicle
VPP	Vertical Profile Projection Curves
XGBoost	eXtreme Gradient Boosting
